# A method for facilitating the seed germination of a mycoheterotrophic orchid, *Gastrodia pubilabiata*, using decomposed leaf litter harboring a basidiomycete fungus, *Mycena* sp.

**DOI:** 10.1186/s40529-017-0214-6

**Published:** 2017-12-08

**Authors:** Kana Higaki, Kento Rammitsu, Yumi Yamashita, Tomohisa Yukawa, Yuki Ogura-Tsujita

**Affiliations:** 1 Faculty of Agriculture, Saga University, 1 Honjo-machi, Saga, 840-8502 Japan; 2 Tsukuba Botanical Garden, National Museum of Nature and Science, 4-1-1 Amakubo, Tsukuba, Ibaraki 305-0005 Japan

**Keywords:** Conservation, *Gastrodia*, Leaf litter-decomposing fungi, Mycoheterotrophic plants

## Abstract

**Background:**

Mycoheterotrophic plants are one of the most difficult plant groups to conserve because they are entirely dependent on symbiotic fungi. Establishment of viable culture systems would greatly aid their conservation. We describe a simple culture system for the mycoheterotrophic orchid, *Gastrodia pubilabiata*, that does not require laboratory facilities. The orchid is symbiotic with leaf-litter-decomposing fungi.

**Results:**

*Gastrodia pubilabiata* seeds were incubated in plastic boxes or glass bottles filled with leaf litter collected from the natural habitat of the species. Seed germination was observed after 35 days and seedling development followed. Fungal isolates from seedlings were identified as Mycenaceae (Basidiomycota), a leaf-litter-decomposing mycorrhizal fungus of *Gastrodia* species.

**Conclusion:**

Our method can be used to conserve endangered mycoheterotrophic plants associated with leaf litter-decomposing fungi efficiently, and can also serve as a model system for physiological and molecular studies of such plants.

**Electronic supplementary material:**

The online version of this article (10.1186/s40529-017-0214-6) contains supplementary material, which is available to authorized users.

## Background

Mycoheterotrophic plants depend on mycorrhizal fungi for their carbon supply throughout their life cycle (Leake 1994). Most such plants are rather rare and seriously endangered, principally because of habitat destruction (Merckx et al. 2013). However, they are also one of the most difficult plant groups to conserve both ex situ and in situ because they are strongly dependent on specific symbiotic fungi. Development of a viable culture system would be of great assistance in terms of efficient plant propagation and subsequent reintroduction into natural habitats. In addition, mycoheterotrophic plants have dust-like seeds with minimal nutrient reserves, and germination occurs mostly underground, rendering it difficult to investigate seed germination ecology in their habitat. Seedling growth in culture would greatly aid studies of seed dormancy, seedling germination, and development. As many biological characteristics of mycoheterotrophic plants remain unknown, a simple culture method would serve as a useful model system for elucidating the physiological and molecular aspects of mycoheterotrophy.

Successful flowering of mycoheterotrophic plants co-cultured with symbionts has been reported for several orchids associated with leaf-litter or wood-decomposing fungi, including *Didymoplexis minor* (Burgeff 1936), *Gastrodia verrucosa* (Tashima et al. 1978), *Gastrodia elata*, (Xu and Guo 2000), and *Epipogium roseum* (Yagame et al. 2007). However, these studies used fungal isolates from mycorrhizal tissues and required laboratory facilities. As a wide range of people, from professional scientists to amateur volunteers, are involved in orchid conservation programs, a versatile method without expensive culture facilities is required. An easy symbiotic culture system without fungal isolation has been successfully achieved for Australian green orchids by incubating orchid seeds and soil from their natural habitat (Brundrett and Ager 2011). Seedlings of *Gastrodia nipponica*, a mycoheterotrophic orchid that is associated with litter-decomposing fungi (Kinoshita et al. 2016), were successfully established by incubating the seeds in a plastic box filled with leaf litter from their habitat (Umata and Nishi 2010).

The mycoheterotrophic orchid *Gastrodia pubilabiata* is principally associated with litter-decomposing fungi in the families Mycenaceae and Marasmiaceae (Kinoshita et al. 2016); both the plant and the fungi can be cultured in vitro. In a previous study, seeds of *G*. *pubilabiata* were successfully germinated and developed into plantlets using in vitro symbiotic culture with a fungus from the mycorrhizal roots of *G*. *verrucosa* (Umata et al. 2000). Asymbiotic seed germination (Godo et al. 2007) and flowering under symbiotic cultivation (Shimaoka et al. 2017; S. Inagaki personal communication) have also been achieved. Seventeen prefectures in Japan list *G. pubilabiata* as an endangered plant (Association of Wildlife Research and EnVision 2013). Furthermore, this orchid is < 30 cm in height and easy to handle, making it a suitable model plant for physiological and molecular studies.

This paper describes a simple method for germinating seeds of *G*. *pubilabiata* using decomposing leaf letter, circumventing the need for laboratory facilities. We also describe the basidiomycete fungus implicated in the germination process. This paper presents a new model system that can be applied to other mycoheterotrophic orchids for conservation purposes.

## Methods

### Sample collection and seed germination

**Fig. 1:**
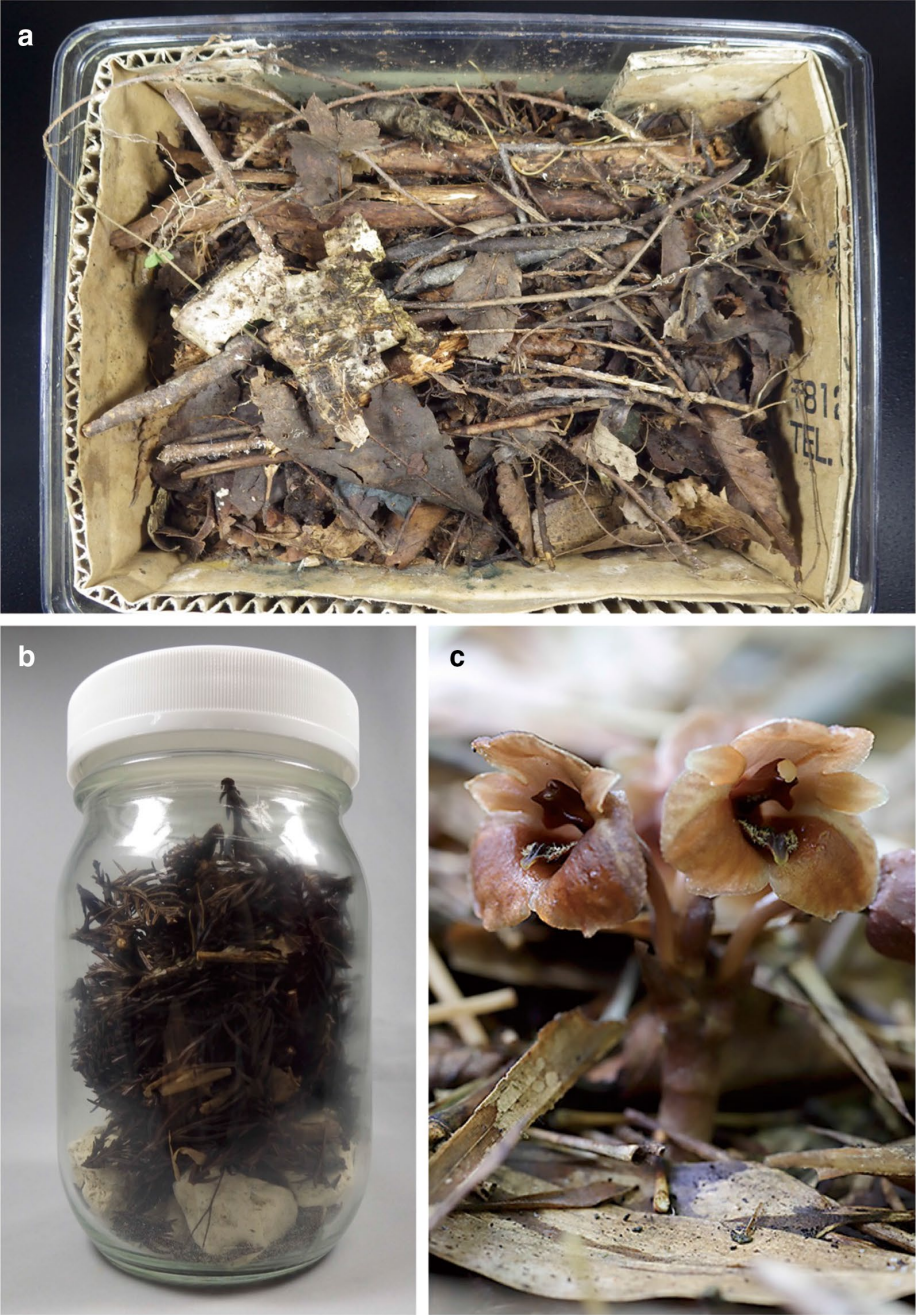
The culture vessels used in this study and flower morphology of *Gastrodia pubilabiata*. Plastic boxes (**a**) and glass bottles (**b**) were filled with leaf litter collected from the natural habitat. **c** A flowering plant in its habitat (photo by T. Yamashita)

Mature seeds from naturally pollinated *G*. *pubilabiata* plants were collected from two sites in Shizuoka Prefecture, Japan, on 22 October 2016. Three and five capsules from different individuals were collected at Sites 1 and 2, respectively. The collected seeds were kept in paper bags for each individual and stored in glass jars with silica gel at ambient temperature for 19 days until use. To verify the difference in leaf litter components, litter was collected from two different habitat types. Site 1 was a broad-leaved forest dominated by *Quercus serrata* mixed with *Zelkova serrata*, *Cinnamomum yabunikkei*, and *Cleyera japonica*, and Site 2 was a monoculture plantation of *Cryptomeria japonica*. Leaf litter was collected at an approximate depth of 5 cm within 2 m of fruiting *Gastrodia* plants at each site and placed in plastic zipper bags (480 × 340 mm). The bags were then carefully packed into a cardboard box for transport by airplane, transported back to the laboratory within 3 days of collection, and kept at ambient temperature in the dark for 19 days until use. Large and small plastic boxes (with lids) and 900- and 450-mL glass bottles with screw caps were sterilized with 70% ethanol and filled with leaf litter from each site (Table 1, Fig. 1). The inner walls of each box were covered with cardboard, and pumice was used to form layers 2 cm thick at the bottom of the bottles. On 9 November 2016, seeds from three and five capsules were bulked and sprinkled over the boxes and bottles, respectively. The vessels were maintained at 25 °C in a growth chamber in the dark. The screw caps of the glass bottles were loosened to allow gas exchange. Non-sterilized tap water was hand-sprayed as a mist when the surface of the leaf litter became dry. Seed germination was evaluated after 35, 65, 70, and 120 days of culture. Seedlings longer than 1 cm were randomly chosen and used for fungal isolation.

**Table 1:** The culture vessels and leaf litter used in this study and the results of seed germination

Vessel number	Size of the vessels	Location of leaf litter collected	Seed germination^b^
Box 1	L260 × W190 × D100^a^	Site 1	++
Box 2	L411 × W337 × D139	Site 1	+
Bottle 3	450 mL	Site 2	+
Bottle 4	450 mL	Site 2	−
Bottle 5	900 mL	Site 2	+

^a^Length (L) × width (W) × depth (D) (mm)

^b^++, a large number of protocorms and seedlings were observed; +, germination was observed; –, no germination

### Fungal isolation

Seedlings were washed three times with sterile water and pelotons in mycorrhizal tissue were teased with needles as described by Rasmussen (1995); these pelotons were cultured on potato dextrose agar (PDA) plates with 50 ppm of streptomycin and tetracycline each. Fungal colonies from single pelotons were transferred to fresh PDA plates for subculture. The fungal isolates obtained in this study were preserved in the Biological Resource Center of the National Institute of Technology and Evaluation (NBRC).

### Molecular identification

DNA was extracted from fungal isolates as described by Izumitsu et al. (2012). PCR and sequencing were performed as described by Ogura-Tsujita and Yukawa (2008). To identify the mycorrhizal fungi, fungal nuclear ribosomal internal transcribed spacer (ITS) regions were amplified using the ITS1F/ITS4 primer pair (White et al. 1990; Gardes and Bruns 1993). As the collected leaf litter might have been contaminated with seeds from other orchid species, the plant species of the seedlings were also identified molecularly using the 17SE/26SE primer pair (Sun et al. 1994), and we confirmed whether the germinated seedlings were consistent with the seeds used in this study. An entire seedling from Box 1 and a tuber larger than 1 cm from Bottle 3 were used for plant identification according to the above method.

## Results

**Fig. 2:**
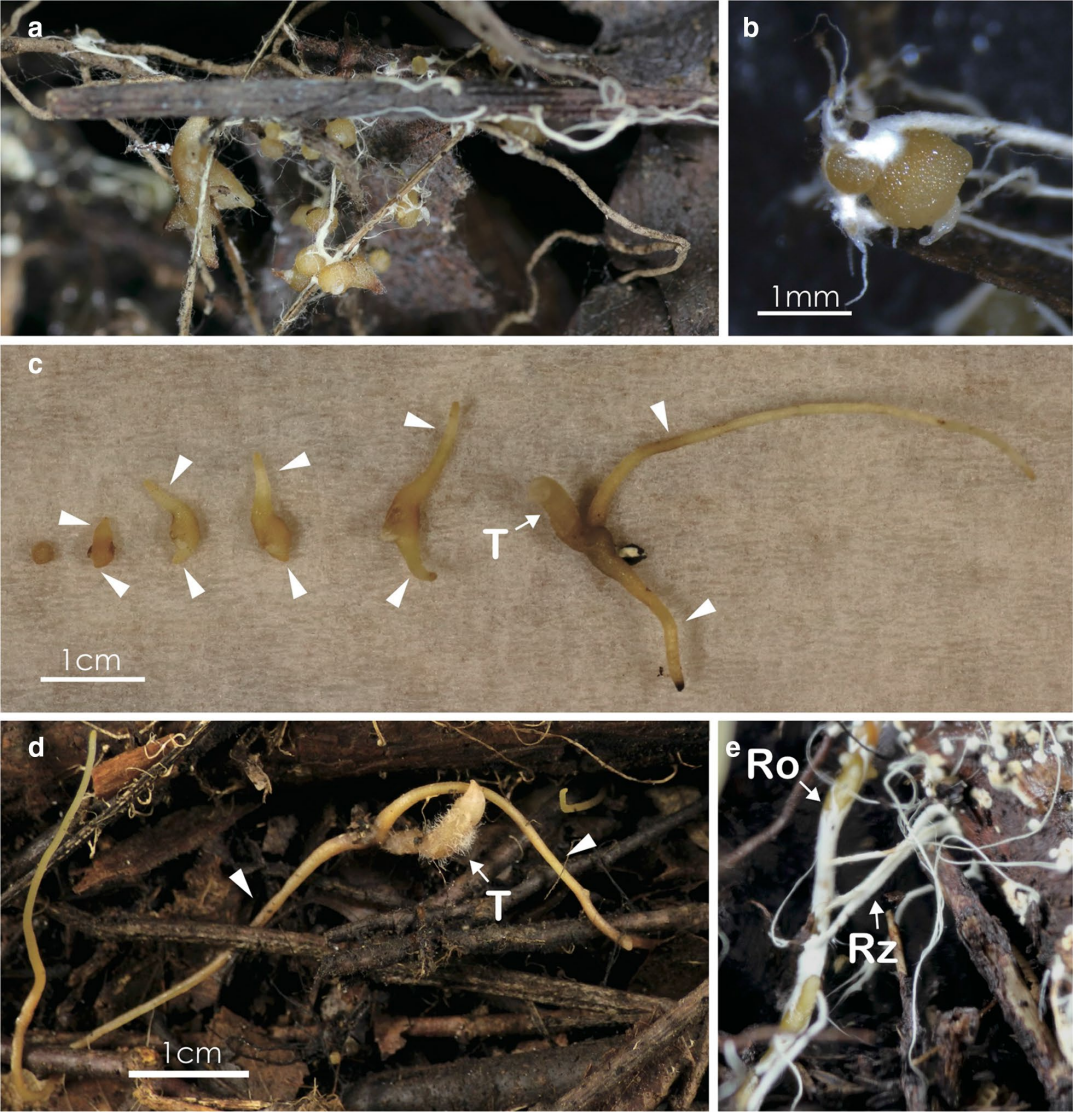
Seed germination and seedling growth of *Gastrodia pubilabiata*. **a**, **b** Protocorms after 35 days of culture. Fungal hyphae and white rhizomorphs are evident around the protocorms; **c** Seedlings after 35 days of culture. Roots (arrows) and tubers (T) are evident; **d** A Box 1 seedling after 65 days of culture. Elongated roots (arrows) and hairy tubers (T); **e** A seedling root with attached fungal rhizomorphs after 120 days of culture. Rhizomorphs (Rz) growing from a dead branch are connected to a mycorrhizal root (Ro)

Seed germination was observed after 35, 65, 70, and 120 days of culture and protocorms were found in all culture vessels, except Bottle 4 (Table 1, Fig. 2). The leaf litter from each of the two sites with different leaf components induced seed germination. Fungal hyphae and rhizomorphs grew around the protocorms (Fig. 2a, b). All developmental stages, from the protocorm to the seedling stage with roots, were observed (Fig. 2c). Each seedling developed two lateral roots, followed by root elongation and tuber development. After 65 days of culture, the tubers developed soft woolly hairs; the longest root was 14 cm (the root of the largest seedling in Box 1; Fig. 2d). Fungal rhizomorphs originating from dead branches (in the leaf litter) were often connected to the roots (Fig. 2e). After 5 months of culture, seedling growth declined as the litter decomposed.

Four and one fungal isolate(s) from a single peloton were obtained from seedlings in Box 1 and Bottle 3, respectively (Table 2). All Box 1 isolates (Isolate ID. F68) were identical in sequence, being 100% homologous to the ITS sequence of the litter-decomposing fungus *Mycena* cf. *abramsii* (KR673481) and 99% homologous to the symbiotic fungus of *G*. *nipponica* (LC013372). The isolate from Bottle 3 (Isolate ID. F69) was 99% homologous to the ITS sequence of the symbiotic fungus of *G*. *pubilabiata* (LC013346), *Mycena plumbea* (JN198391), and a *Mycena* species that induces seed germination in *G*. *elata* (FJ785523). The rhizomorph connecting the mycorrhizal roots of a seedling in Bottle 3 had a sequence identical to that of F69. Isolates F68 and F69 shared similar morphological characteristics and grew slowly on PDA (Additional file 1: Figure S1). The colonies were white and relatively flat, with diffuse to filamentous edges, and their diameters were approximately 5 cm after 2 weeks.

**Table 2:** The results of molecular identification of the fungal isolates from *G. pubilabiata* seedlings

Isolate	NBRC no.^a^	Accession no. of ITS sequence	Closest mach in GenBank	Identities
F68	NBRC113007	LC314114	*Mycena abramsii* voucher KA12-0434 (KR673481)	607/607 (100%)
			Mycorrhizal fungus of *G. nipponica* (LC013372)	650/659 (99%)
F69	NBRC113008	LC314115	Mycorrhizal fungus of *G. pubilabiata* (LC013346)	642/646 (99%)
			*Mycena plumbea* (JN198391)	641/645 (99%)
			*Mycena* sp. MKACC51882 (FJ785523)	640/645 (99%)

^a^All isolates were deposited to the Biological Resource Center of the National Institute of Technology and Evaluation (NBRC)

## Discussion

Seeds of *G*. *pubilabiata* germinated in four of the five culture vessels used in this study (Table 1), and germination was achieved within 35 days after sowing. There were no differences in seedling establishment among the four types of culture vessel, although the culture in Box 1 resulted in the large number of protocorms (> 50 individuals), while the largest tuber was observed in Bottle 3. The leaf litter components also did not affect seed germination. Fungal rhizomorphs growing from dead leaves and tree branches were often connected to protocorms and roots (Fig. 2a, b, e), indicating that litter-decomposing fungal rhizomorphs associate with *G*. *pubilabiata* to transfer carbon from leaf litter to the seedlings. Long-term seedling culture was not achieved in this study. As the leaf litter in the box was broken down into small pieces and white vigorous rhizomorphs turned yellowish, it seems likely that the fungal growth resources became depleted over time. Continuous replenishment of leaf litter may be required for long-term seedling culture. Successful fungal isolation from seedlings indicates that the culture system established in this study could be useful as a seed baiting technique to detect symbiotic fungi from orchid habitats, as shown for terrestrial green orchids (Brundrett et al. 2003). In addition to the leaf litter culture, 70 packets containing orchid seeds were buried for 1 year in the same habitats, but only five seedlings (< 5 mm) were obtained from these packets (data not shown). It seems likely that the leaf litter culture may serve as a propagation method for *G. pubilabiata*, although additional replicates are required.

The molecular data imply that the Mycenaceae promote seed germination and seedling growth. The Mycenaceae family constitutes prominent fungal partners of *G*. *pubilabiata* and two other *Gastrodia* species, *G*. *confusa* and *G*. *nipponica* (Ogura-Tsujita et al. 2009; Kinoshita et al. 2016). *Mycena osmundicola* has been shown to induce germination of *G*. *elata* seeds (Xu and Guo 1989). In our study, the fungal isolates were highly homologous to the symbiotic fungi of *G*. *nipponica* and *G*. *elata*, indicating that the symbiotic *Mycena* species that we detected may be associated with several *Gastrodia* species. Furthermore, the F69 isolate was 99% homologous to a symbiotic fungus from adult *G*. *pubilabiata* plants, suggesting that a single *Mycena* species may be present throughout the entire life cycle of *G*. *pubilabiata*, in contrast to the situation in *G*. *elata*, in which the fungal partner shifts from *Mycena* to *Armillaria* as seedling development proceeds (Xu and Guo 2000).

## Conclusions

We found that *G*. *pubilabiata* seeds germinated in culture vessels filled with leaf litter collected from the natural habitat; fungal isolation was unnecessary. Our system may assist ex situ conservation programs, allowing seedling culture, propagation, and reintroduction of *Gastrodia* and other mycoheterotrophic orchids associated with litter-decomposing fungi such as *Didymoplexis* (Burgeff 1932). Furthermore, our method may serve as a model system for observing seedling development and physiological and molecular studies of mycoheterotrophic plants.

## Additional file

**Additional file 1: Figure S1.** Morphological characteristic of the fungal isolate F69.
