# Supplementary material for: A method for facilitating the seed germination of a mycoheterotrophic orchid, Gastrodia pubilabiata, using decomposed leaf litter harboring a basidiomycete fungus, Mycena sp
Source: Bot Stud. 2017 Dec 8;58:59. doi: 10.1186/s40529-017-0214-6 (PMC5722783; doi:10.1186/s40529-017-0214-6)
Supplement: Supplementary file 1 — Additional file 1: Figure S1. Morphological characteristic of the fungal isolate F69. [file 40529_2017_214_MOESM1_ESM.pdf]

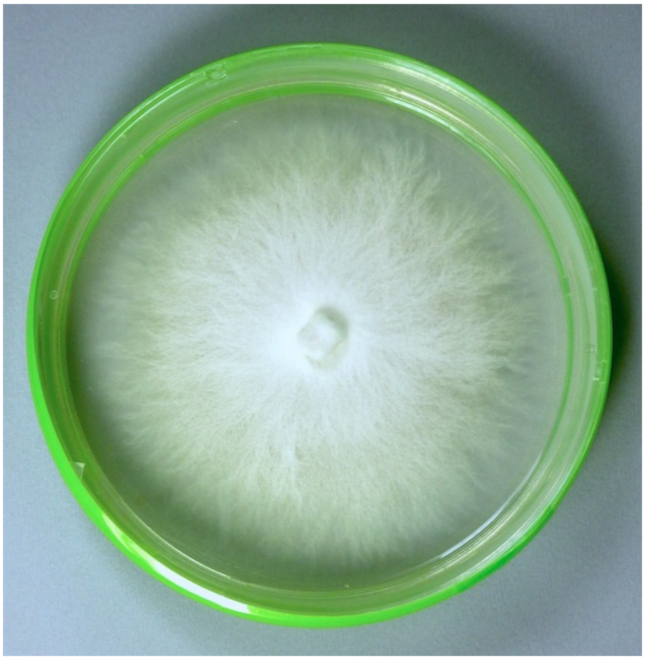

Figure S1. Morphological characteristic of the fungal isolate F69 on PDA plate after three weeks culture.
